# Pantoprazole Sodium Loaded Microballoons for the Systemic Approach: *In Vitro* and *In Vivo* Evaluation

**DOI:** 10.15171/apb.2017.055

**Published:** 2017-09-25

**Authors:** Pravin Gupta, Manish Kumar, Darpan Kaushik

**Affiliations:** ^1^Department of Pharmaceutics, Agra Public Pharmacy College, Agra (U.P.), India.; ^2^Department of Pharmaceutics, Maharishi Markandeshwar College of Pharmacy, Maharishi Markandeshwar University, Mullana-Ambala (Haryana), India.; ^3^Department of Pharmaceutical Chemistry, Agra Public Pharmacy College, Agra (U.P), India.

**Keywords:** Eudragit, Gastro retentive drug delivery, Gastric residence time, Non-effervescent, Optimization

## Abstract

***Purpose:*** Various floating and pulsatile drug delivery systems suffer from variations in the gastric transit time affecting the bioavailability of drugs. The objective of the study was to develop Pantoprazole Sodium (PAN) microballoons that may prolong the gastric residence time and could enhance the drug bioavailability.

***Methods:*** Microballoons were prepared using Eudragit^®^L100 by adopting emulsion solvent diffusion method with non-effervescent approach, in vitro studies were performed and the in vivo evaluation was carried out employing ethanol induced ulceration method. Optimization and validation were carried out through Design Expert^®^ software.

***Results:*** The results demonstrate an increase in percentage yield, buoyancy, encapsulation efficacy and swelling. Particles were in the size range 80-100 µm following zero order release pattern. SEM study revealed their rough surface with spherical shape, internal cavity and porous walls. DSC thermo gram confirms the encapsulation of drug in amorphous form. Significant anti ulcer activity was observed for the prepared microballoons. The calculated ulcer index and protection were 0.20±0.05 and 97.43 % respectively for LRS-O (optimized formulation).

***Conclusion:*** This kind of pH dependent drug delivery may provide an efficient dosage regimen with enhanced patient compliance.

## Introduction

Pantoprazole Sodium (PAN) has high solubility and high permeability so it is a difficult task to restore its efficacy. Due to its low bioavailability (77%) and short half-life (1 h) its administration is preferred through intravenous (IV) route but for the non-invasive therapy it is given through oral route as multiple unit dosage form such as floating microspheres that efficiently reduces the dosing frequency.^1^

In previous reported works it has been formulated as gastro-resistant multiple unit systems by emulsion solvent diffusion and spray-drying methods using Eudragit^®^S100.^2^ Micro particles formulated through emulsion solvent diffusion method were larger in size and were able to stabilize 61% of PAN content after acid exposure. Baclofen loaded microballoons with a hollow core floated for more than 10 h.^3^ The *in-vivo* anti-ulcer activity as in case of Nizatidine microballoons confirms protection of gastric mucosa against ethanol induced ulceration, also the gastric residence time and bioavailability of drug in the gastrointestinal tract could be prolonged.^4^

Effervescent method was reported with the adverse effects of violent gas generation, disintegration of dosage form, burst release, dose dumping and produces alkaline microenvironment.^5^ In our study microballoons intended for extended release were prepared by non-effervescent technique using magnesium stearate, Eudragit^®^L100, Eudragit^®^RS100 and analyzed simultaneously.

## Materials and Methods

### Materials

Pantoprazole Sodium (PAN) was obtained from Akum Drugs (Haridwar, India). Eudragit^®^L100 and Eudragit^®^RS100 were received as a generous gift from Evonic industries (Mumbai, India). All other chemicals used were of analytical grade.

### Preparation of PAN loaded microballoons

For the preparation Eudragit^®^L100 and Eudragit^®^RS100 (600-900 mg) were dissolved using equal proportions (each 8 ml) of ethanol and dichloromethane, and a suitable plasticizer DBT (Dibutylphthalate, 20% w/v) was added for enhancing the solubility of polymers. Magnesium Stearate (2.5-5 % w/w) solubilized in warm ethanol was added. PAN (40 mg) was dissolved separately in distilled water containing sodium chloride (0.09 g) and it was slowly incorporated in to the above polymer solution with continuous stirring for 1 h. This drug-polymer dispersion was slowly added to PVA (polyvinylalcohol) aqueous solution (0.75% w/v in 200 ml distilled water) containing sodium citrate (1% w/v) to form oil in water type of emulsion and was stirred for 1 h (300 rpm, 40°C).^6^ Ethanol and dichloromethane evaporates from the dispersed droplets and they solidify. Then they were filtered, washed thrice with distilled water and kept aside for drying at room temperature until constant weight.^7,8^

### Experimental Design

Design-Expert^®^9.0.3 software (Stat-Ease Inc., USA) was used in optimizing 2^3^ full factorial designs (FFD) for the LRS (code) formulations, as shown in Table 1.^9^

**Table 1:** Full factorial design layouts (2^3^) for LRS formulations in phosphate buffer pH 6.8.

**Formulation code**	**Independent variables (factors,** ***X*** )			**Dependent variables (responses,** ***Y*** )		
	**Magnesium stearate** ***(X***_1_ ***, % w/w)***	**Eudragit** ^®^ ** L100** ***(X***_2_ ***, mg)***	**Eudragit** ^®^ **RS100** ***(X***_3_ ***, mg)***	^ a,d^ **B** ***(Y***_1_ ***, %)***	^ b,d^ **EE** ***(Y***_2_ ***, %)***	^ c,d^ **CDR12 h** ***(Y***_3_ ***, %)***
**LRS-1**	2.5 (-1)	600(-1)	600(-1)	28.97±0.021	10.88±0.045	75.05±0.017
**LRS-2**	5.0 (+1)	600(-1)	600(-1)	78.88±0.043	71.12±0.008	99.50±0.015
**LRS-3**	2.5 (-1)	900(+1)	600(-1)	41.03±0.024	26.67±0.021	95.92±0.026
**LRS-4**	5.0 (+1)	900(+1)	600(-1)	75.59±0.011	77.09±0.012	71.55±0.018
**LRS-5**	2.5 (-1)	600(-1)	900(+1)	*collapsed*	*collapsed*	*collapsed*
**LRS-6**	5.0 (+1)	600(-1)	900(+1)	46.87±0.025	40.71±0.046	66.54±0.072
**LRS-7**	2.5 (-1)	900(+1)	900(+1)	61.05±0.034	30.86±0.063	64.08±0.084
**LRS-8**	5.0 (+1)	900(+1)	900(+1)	88.46±0.009	62.13±0.031	77.01±0.064

(+1) = higher values; (-1) = lower values; ^a^ B % = percentage buoyancy; ^b^EE % = percentage encapsulation efficiency; ^c^CDR12 h % = cumulative percentage drug release over 12 h; ^d^Mean ± S.D.: n = 3

### Evaluation of PAN Loaded Microballoons

#### Determination of Encapsulation Efficacy (EE %)

Microballoons equivalent to 40 mg of PAN were weighed, crushed in a mortar and dissolved in 100 ml phosphate buffer pH 6.8 and the filtrate concentration was determined.

The percentage encapsulation was determined using equation 1:^10^

Equation 1 $$\text{EE \%}=\frac{\text{Calculated drug content }\left(\text{x}\right)}{\text{Theoretical drug content}}\;\times 100$$

#### Determination of Percentage Swelling (Ps)

The percentage swelling was found out by weighing 50 mg of dried microballoon and immersed in phosphate buffer pH 6.8 (100 ml, 37±0.5°C) in a beaker, kept over a magnetic stirrer maintained at 100 rpm. The percentage swelling was calculated in triplicate by equation 2: ^11^

Equation 2 $$\text{Ps}=\left\{\frac{\text{Ws}-\text{Wd}}{\text{Wd}}\right\}\times 100$$

Where, *Ws* is weight of swollen and *Wd* is the weight of the dried microballoons.

#### Test of Buoyancy (B %) 

The floating efficiency was determined by dispersing 50 mg of dried microballoon separately in a 250 ml beaker containing phosphate buffer pH 6.8 (100 ml, 37± 0.5°C) with paddle rotation of 100 rpm. After 12 h they were collected, dried and weighed. Weight of floated (W_F_) and those settled down (W_NF_) were found out and the percentage buoyancy was estimated using equation 3:^12^

Equation 3 $$\text{B}\%=\left\{\frac{{\text{W}}_{\text{F }}}{{\text{W}}_{\text{F }}+{\text{W}}_{\text{NF}}}\right\}\times 100$$

#### Optimization and Validation of Design

Further optimization of best design was carried out using Design Expert^®^ software.^13^ Validation was done by generating polynomial equations for each response consisting of interactive and polynomial terms:

Equation 4 $$y=b_{0}+\;b_{1}X_{1}+b_{2}X_{2}+b_{3}X_{3}+b_{12}X_{1}X_{2}+b_{13}X_{1}X_{3}+b_{23}X_{2}X_{3}+b_{123}X_{1}X_{2}X_{3}$$

Where *b*_0_, the intercept represents the arithmetic mean; *b*_1_, *b*_2_, *b*_3_, *b*_12_, *b*_13_, *b*_23_ and *b*_123_ are the main effects calculated by adding or subtracting the obtained responses, *Y*. The interaction effects: *X*_1_*X*_2_, *X*_1_*X*_3_, *X*_2_*X*_3_ and *X*_1_*X*_2_*X*_3_ were calculated same as that of the main effects.^14^

An extra check point formulation was formulated and the significance of the model design was estimated (*p*<0.05) using one-way ANOVA method. The actual and the predicted responses were calculated to find out the percentage error:

Equation 5 $$\text{Percentage error }\left(\text{\%}\right)=\frac{\left(\text{predicted value}-\text{experimental value}\right)}{\text{predicted value}}\times 100$$

#### Characterization of PAN Loaded Microballoons

#### Particle Size Determination

Sizes of 200 microballoons were determined and their numbers are tabulated in each size range and the percentage in each range was estimated using following equation. The particle size distribution was also found out by plotting percentage in each range against the size range:

Equation 6 $$\text{\% in each range}=\frac{\text{Number of particles}}{200}\times 100$$

#### Scanning Electron Microscopy (SEM)

The SEM analysis was performed by JEOL 5400, Kyoto, Japan and the micrographs were obtained at magnifications such as 1 x, 150 x and 500 x.

#### Differential Scanning Calorimetry (DSC)

DSC analysis (NETZSCH DSC 200F3 240-20-427-L) of PAN, Eudragit^®^L100, Eudragit^®^RS100, physical mixtures (Magnesium stearate + Eudragit^®^L100 + Eudragit^®^RS100), and the optimized formulation (LRS-O) were performed by sealing about 2-3 mg of the samples in aluminum pans and calibrated using indium.

#### Stability study

The optimized formulation (LRS-O) was stored in stability chamber at 40 ± 2°C / RH 75 ± 5% for 6 months and periodically evaluated for physical changes, percentage buoyancy and percentage encapsulation efficacy.

#### In vitro release study

The drug release from formulation code LRS-2, LRS-O and Pantop-40 (Aristo pharmaceutical Pvt. Ltd. Mumbai, India) were performed in phosphate buffer pH 6.8. Microballoons equivalent to 40 mg of PAN was weighed and placed in 900 ml medium with continuous stirring (37°C, 50 rpm) and the samples were analyzed after fixed intervals of 1 h up to 12 h in triplicate.^15^

#### Drug release kinetics

Drug release profiles were fitted in to various kinetic models in order to find out the mechanism involved. Regression equations were generated for zero-order, first-order, Higuchi and korsmeyer-peppas model by plotting Time (T) Vs Cumulative percentage drug release (CDR), T Vs log CDR, √T Vs CDR and log T Vs log %CDR respectively.

#### In vivo Anti-ulcer Activity 

Male wistar rats were divided in to five groups, each group comprises of eight animals weighing between 150-200 g. They all were deprived of food for a period of 18 h and allowed to have access of water and were kept in separate cages to prevent coprophagy. Except the normal group all other groups receives ethyl alcohol (5 ml. kg^-1^) orally. After 1 h of ethyl alcohol dose they receive the respective doses of samples, as shown in Table 2. After 2 h they were anesthetized with diethyl ether (1.9%) in desiccators, this concentration was produced with 0.08 ml (80 micro liters) per liter volume of a container, then sacrificed, the stomach was removed, cut along the greater curvature and after rinsing with distilled water stretched over thermo coal with mucosal side up and are examined for gastric lesions. Ulcer Indexes (UI) were calculated using equation 7:^16^

Equation 7 $$UI=\frac{10}{x}$$

Where, *x* represents the total mucosal area divided by the total ulcerated area.

Ulcer index graph was made using graph pad prism^®^version 6.05 with MEAN ± SEM.

**Table 2:** Groups selected and the administered doses for the *in vivo* anti-ulcer activity.

**Groups**	**Administered samples**	**Route**
**Normal**	1 % gum acacia	orally
**Control**	Ethyl alcohol (5 ml. kg^-1^)	orally
**Standard-1**	Sodium bicarbonate solution (4.2 %)	orally
**Standard-2**	PAN dissolved in distilled water (2 mg. ml^-1^)	IV
**Treatment**	LRS-O microballoons (equivalent to 2 mg.ml^-1^ of PAN) dispersed in 0.5 ml Gum Acacia	Orally

IV: Intravenous, PAN: Pantoprazole Sodium, LRS-O: Optimized formulation

## Results and Discussion

### Preparation and optimization of PAN loaded microballoons

In phosphate buffer pH 6.8 maximum swelling and the amount of medium uptake were 90±0.011%, 0.045±0.004 g/g. This is attributed due to higher concentration (5% w/w) of magnesium stearate that in turn provides hydrophobicity to the formulation thus reduces their density and provides buoyancy.^17^ It suggest for the retarded drug release due to the blockage of pores in the polymer matrix.^18^ Percentage buoyancy was between 28.97-88.46% contributed due to reduced density of the polymers moreover the solvent evaporation provides a hollow cavity inside. The internal cavity was filled with the medium in floating condition because of porous boundary wall. Higher encapsulation efficacy (10.88-77.09%) was due to higher concentration of magnesium stearate and Eudragit^®^L100. Eudragit^®^L100 solubilizes above pH 6 while Eudragit^®^RS100 makes the formulation porous and magnesium stearate gave rough surface that readily releases the drug that was detected spectrophotometrically.

We have elucidated the main and the interaction effects of independent variables over the responses through response surface method using Design Expert^®^9.0.3 software. From the ANOVA results for the dependent responses B%, EE% and CDR12 h%, the model equation for B % showed that coefficients *b*_3_, *b*_12_ and *b*_13_ has no static significance (*p*>0.05) with the model *F-*value of 380.63 and *R*^2^ value of 0.9970, the model equation for EE % has all coefficients significant with model *F*-value of 51186 and *R*^2^ value of 0.9989, whereas for the model equation for CDR12 h% it was evident that all the coefficients has static significance with the model *F-* value of 380.63 and *R*^2^ value of 0.9990. For model simplification the non-significant terms *p*>0.05 were eliminating from all polynomial equations, so the final equation becomes:

Equation 8 $$B\%\;\left(Y_{1}\right)=60.12+22.67X_{1}+15.91X_{2}+13.41X_{2}X_{3}-0.58X_{1}X_{2}X_{3}$$

Equation 9 $$EE\%\;\left(Y_{2}\right)=45.63+26.09X_{1}+10.57X_{2}-7.43X_{3}-2.75X_{1}X_{2}-5.52X_{1}X_{3}+4.36X_{2}X_{3}+\;0.05X_{1}X_{2}X_{3}$$

Equation 10 $$CDR12\;h\;\%\;\left(Y_{3}\right)=78.52+11.36X_{1}+9.63X_{2}-19.19X_{3}-14.63X_{1}X_{2}+11.34X_{1}X_{3}+11.66X_{2}X_{3}$$

Above equations confirm that by increasing the concentration of independent variable-1 (magnesium stearate) the responses could be increased by 22.67% (B%), 26.09% (EE%) and 11.36% (CDR 12 h%) respectively. In our previous work the response surface plots for B% gave an increase in response with increase of both magnesium stearate (*X*_1_) and Eudragit^®^L100 (*X*_2_), whereas EE% predicts an increase of response with an increase of both magnesium stearate (*X*_1_) and Eudragit^®^L100 (*X*_2_) and decrease of Eudragit^®^RS100 (*X*_3_), moreover the plot for CDR12 h% confirms an increase of response with increasing magnesium stearate (*X*_1_), Eudragit^®^L100 (*X*_2_) and decreasing Eudragit^®^RS100 (*X*_3_).^19^

Desired responses were obtained using numerical optimization technique that reduces the number of trials.^20^ Optimized formulation (LRS-O) showed buoyancy of 78.88±0.23%, entrapment efficiency of 71.12±0.04% and drug release in 12 h of 99.50±0.08% with smaller error values (0.617, -0.042 and 0.490), the percentage error was found out to be of low magnitude, which validates the design.

### Particle size analysis

The method demonstrates increase in particle size with increase in the polymer ratios. Maximum particles (27.5±0.07%) were found in the range 80-100 µm, larger size was due to the higher cross-linking effect of DBT. The results of stability study of the optimized formulation carried out for a period of six months showed no physical change among themselves. The ANOVA values for *F* at 5% level of significance for B% and EE % was 16.29 and 15.16. Since the calculated value for *F* was found to be less than the tabulated (*F*_Tab_=225), the difference was not significant and we conclude that the means do not differ among themselves only a slight decrease in buoyancy and encapsulation efficacy was observed that was insignificant.

### Scanning Electron Microscopy

**Figure 1:**
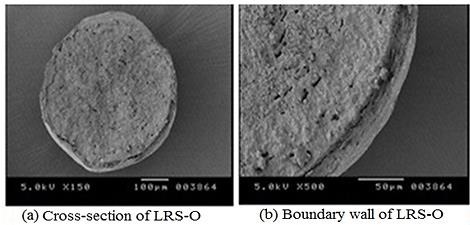
Scanning electron micrographs of optimized formulation (LRS O) (a) Cross-section (b) Boundary wall.

The micrograph as shown in Figure 1 was found to have rough surface due to higher rate of cross-linking between polymers. Spherical shape may be attributed to the reduction in surface free energy due to surface tension. Internal hollow cavity was due to evaporation of volatile solvent mixture. The porosity or channels on the boundary wall was due to the porous nature of the polymer Eudragit^®^RS100 and also due to channeling effect of sodium chloride.

### Differential Scanning Calorimetry

**Figure 2:**
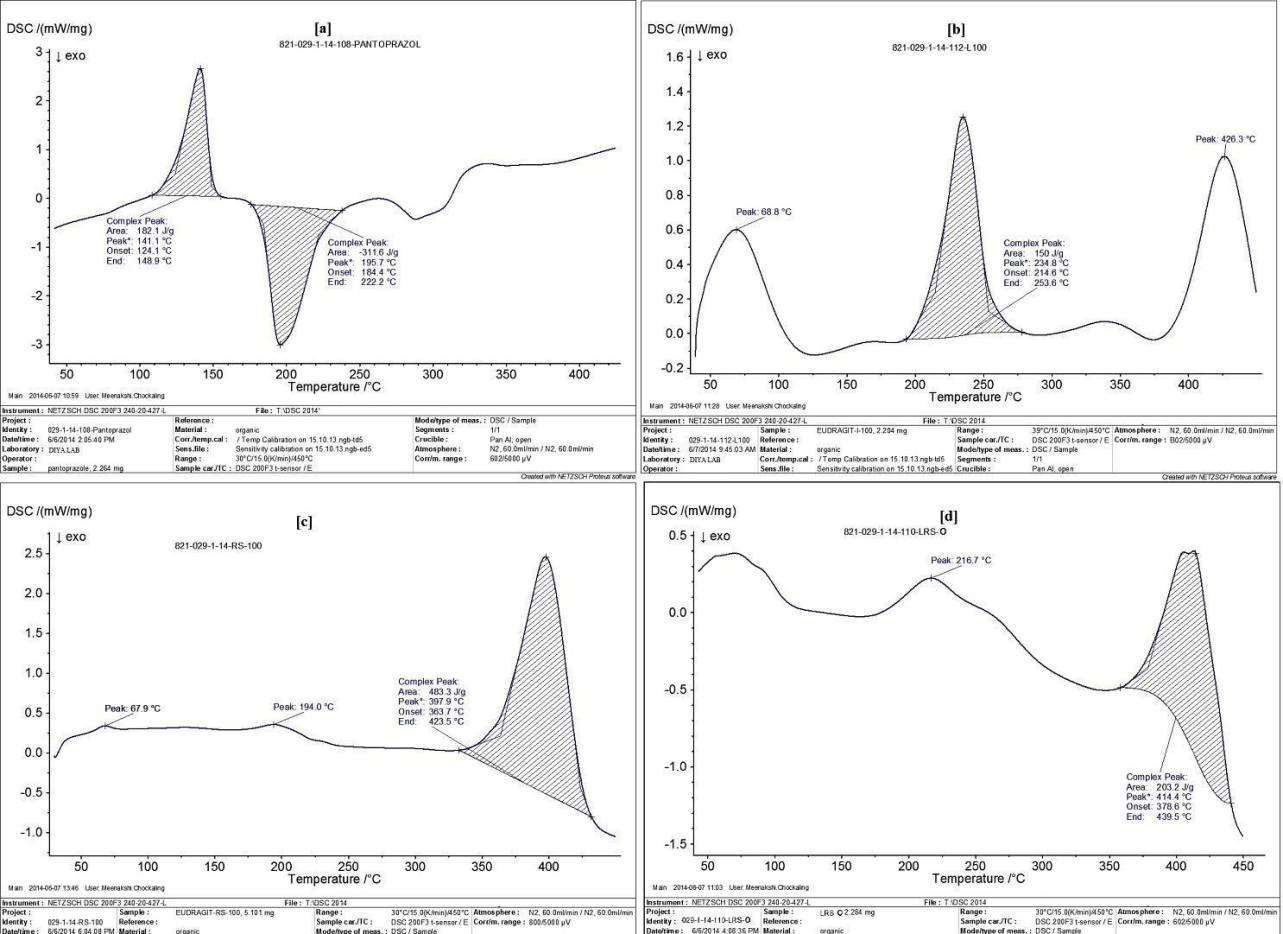
Differential Scanning Calorimetry thermogram of (a) Pantoprazole Sodium (PAN), (b) Eudragit^®^L100, (c) Eudragit^®^RS100, (d) Formulation LRS O.

In the formulation Eudragit^®^L100 presented two endothermic peaks at 68.8and 426.3°C and a complex peak at 234.8°C. Thermo gram of physical mixture containing Magnesium stearate, Eudragit^®^L100 and Eudragit^®^RS100gave a complex endothermic peak at 102.9 °C and another two endothermic peaks at 246.6and 331.4 °C. In Figure 2 optimized formulation (LRS-O) presents an endothermic peak at 216.7°C and a complex endothermic peak at 414.4°C. The higher peak values may be due to increased cross linking between polymers, thus shifts the glass transition towards higher temperature. Moreover DBT shifts the glass transition temperature towards lower values as in case of magnesium stearate.

### In vitro release

**Figure 3:**
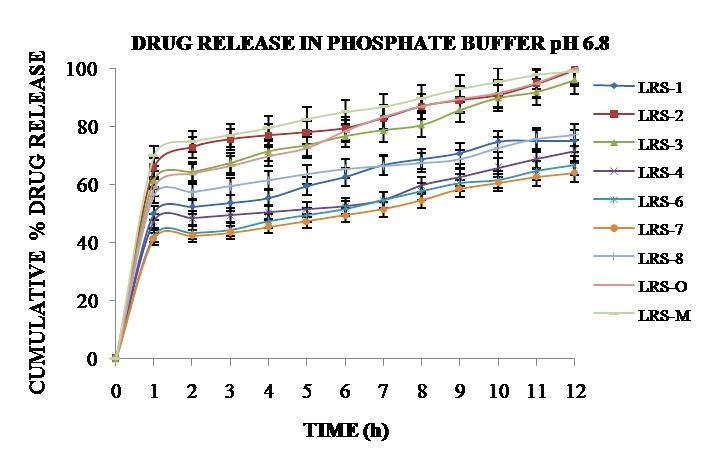
Cumulative percentage *in vitro* drug release profiles of the Best (B), Optimized (O) and Marketed (M) formulation in phosphate buffer pH 6.8 for 12 h (37 ± 0.5 °C, 300 rpm).

Initial release of PAN from microballoons were higher and after some time lag it was sustained as polymer matrix becomes denser, thus the diffusion path length increases that favors in prolonged drug release characteristics. In phosphate buffer pH 6.8, LRS-O (optimized formulation) gave maximum release of 99.50±0.015% over 12 h studies when compared to LRS-M (marketed formulation) of 98.89 ± 0.05%, shown in Figure 3. Eudragit^®^RS100 provides porous nature due to presence of ammonium groups whereas sodium chloride initiates via its channeling effect.^21^ Magnesium stearate used was of low bulk density and hydrophobic in nature thus enhances the floating ability.^22^

The observed release mechanism for formulations i.e. LRS-2, LRS-O and LRS-M was zero order. Initially the polymer chain was relaxed that facilitates the drug release, after sometime pH dependent polymers swells and forms closely packed networks which hinders further entry of dissolution medium thus results in sustained drug release characteristics.^23^

### In vivo anti-ulcer activity

**Figure 4:**
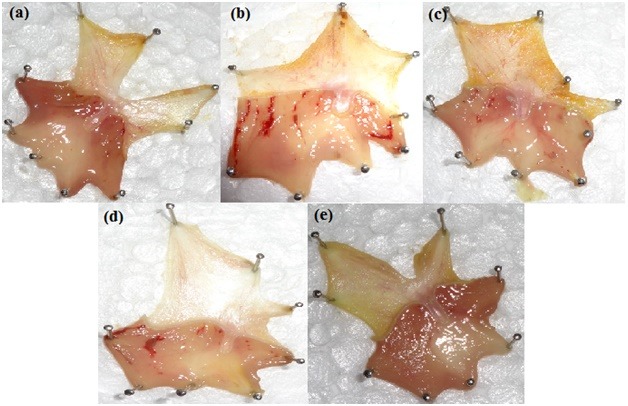
Inner stomach wall of animals treated with: (a) 1 % gum acacia (b) ethyl alcohol, (c) sodium bicarbonate aqueous solution, (d) standard Pantoprazole Sodium solution, (e) LRS O aqueous suspension.

**Figure 5:**
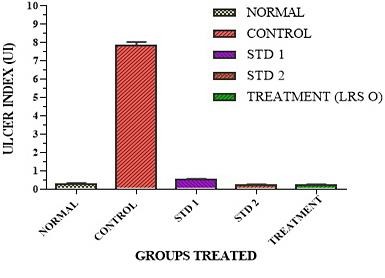
Ulcer Indexes (UI) for various treated groups.

The stomachs of normal group were devoid of any gastric lesions whereas the control group was full of hemorrhagic streaks due to stasis in the mucosal walls. Gastric lesions caused due to ethanol were attributed to the formation of free radical that in turn results in lipid per oxidation product formation.^24^ Treatment with standard-1, shown to have red coloration while the standard-2 treatment gave spot ulcers. On the other hand when administered with treatment dose, with LRS-O, as shown in Figure 4 demonstrates complete removal of hemorrhagic streaks. Similar study for stomach specific delivery was performed using Eudragit^®^E100.^25^ ANOVA analysis confirms that the Ulcer Index values for the treatment groups were lower than that of the standard groups with P < 0.001, as shown in Figure 5.

## Conclusion

PAN loaded microballoons were found to be efficient in ulcer healing and could be delivered through oral route. The delayed release dose can maintain the effective therapeutic level of the drug. This activity may be advantageous for the delivery of acid labile drugs having high solubility and poor absorption in the GIT.

## Acknowledgments

The author wish to thank Akums Drugs (Haridwar, India) and Evonic industries (Mumbai, India) for providing gift samples of Pantoprazole Sodium, Eudragit^®^E100, Eudragit^®^L100 and RS100. We are also thankful to Diya lab, Mumbai for performing the DSC studies.

## Ethical Issues

The entire study was performed in accordance with the guidelines given by “Institutional Animal Ethics Committee” (IAEC) with approval number - SMGI/SMIP/IAEC/2015/011.

## Conflict of Interest

Authors declare no conflict of interest in this study.
